# Radium levels in Brazil nuts: A review of the literature

**DOI:** 10.1111/nbu.12717

**Published:** 2024-11-03

**Authors:** Christian Koeder, Markus Keller

**Affiliations:** ^1^ Institute for Prevention and Cancer Epidemiology, Faculty of Medicine University of Freiburg Freiburg Germany; ^2^ Research Institute for Plant‐Based Nutrition (IFPE) Biebertal Germany

**Keywords:** *Bertholletia excelsa*, Brazil nuts, cancer, radiation, radioactivity, vegetarian

## Abstract

Brazil nuts are well known for their extraordinarily high selenium content. For this reason, they are frequently recommended as a kind of natural selenium ‘supplement’, particularly for certain population groups such as vegetarians and vegans in regions with low soil selenium levels. Typically, an intake of one or two Brazil nuts per day is recommended. Brazil nuts, however, also stand out from other nuts in terms of their high (albeit highly variable) radium content. The radium isotopes Ra‐226 and Ra‐228 emit alpha‐ and beta‐radiation, with this type of radiation being particularly harmful when ingested. Consequently, it is important to consider radium levels in Brazil nuts before formulating recommendations for a long‐term, daily intake of these nuts. To date, however, no comprehensive overview of radium levels in Brazil nuts has been published. Therefore, a literature review without time or language restrictions was conducted, including unpublished original data from Germany. The literature review (including the German data) indicated mean Ra‐226 and Ra‐228 levels of 49 (range: 17–205) mBq/g and 67 (range: 12–235) mBq/g, respectively. Assuming a consistent daily intake of one or two Brazil nuts, this would result in an effective dose of ~88–220 μSv/year. This level of exposure appears to be neither clearly harmful nor clearly harmless. As increased radioactivity exposure (at least at higher doses) is associated with increased cancer risk, randomised controlled trials assessing the effect of Brazil nuts on cancer risk biomarkers are needed.

## INTRODUCTION

Brazil nuts (*Bertholletia excelsa*) are a unique food whose nutritional composition is highly complex as are the sustainability aspects (e.g. the preservation of the Amazon rainforest and indigenous rights) uniquely associated with this particular food (Ribeiro et al., 2014). Brazil nuts are best known for their exceptionally high selenium content. Because of this, one or two nuts a day are frequently recommended as a natural way to add selenium to one's diet (Koeder & Perez‐Cueto, 2024). However, consuming more than two Brazil nuts per day (assuming a selenium content of 5–72 μg/g dry weight) is not recommended as this may already exceed the European tolerable upper intake level for this mineral (255 μg/day) (EFSA et al., 2023). Nevertheless, consuming a good plant source of selenium appears to be particularly useful for those who considerably reduce or avoid the intake of animal‐source foods, for example, vegetarians and vegans (Koeder & Perez‐Cueto, 2024), with such dietary patterns being increasingly popular. Animal products are often richer in selenium compared with plant protein sources (legumes, grains, nuts and seeds) both because the animals' metabolism strives to maintain adequate selenium levels and because animal feed is typically fortified with selenium (Combs, 2001). While Brazil nuts are exceptionally selenium‐rich, a potential concern is that they frequently are also high in radium isotopes which expose the person who eats these nuts to radioactivity (Martins et al., 2012; Parekh et al., 2008; Turner et al., 1958).

Brazil nuts frequently contain relatively high levels of radium, a naturally occurring radioactive metal, with the most common radium isotopes being Ra‐226 and Ra‐228 (Martins et al., 2012). Ra‐226 and Ra‐228 are radionuclides, that is, they are unstable isotopes and emit ionising radiation (i.e. radioactivity) (Martins et al., 2012). The variability of radium levels in Brazil nuts appears to be high, however, including in nuts of the same batch (Gabay & Sax, 1969).

Ra‐226 emits mostly alpha radiation (Straub et al., 2018; USNRC, 1999) and has a physical half‐life of 1600 years (Brugge & Buchner, 2012; USNRC, 1999) while Ra‐228 almost exclusively emits beta radiation and has a physical half‐life of 5.75 years (Abergel et al., 2022; Brugge & Buchner, 2012; USNRC, 1999). Unlike gamma radiation (which can easily penetrate barriers), alpha and beta radiation are particularly damaging if the radiation source is ingested (such as with food or water (WHO, 2022)) or inhaled (US EPA, 2023). This indicates that eating Brazil nuts daily may be harmful (Brugge & Buchner, 2012). Non‐dietary exposure to radium has been associated with anaemia, tooth loss, lower attained height in children (Brugge & Buchner, 2012), impaired bone health (Abergel et al., 2022; Brugge & Buchner, 2012; Martinez et al., 2022) and increased cancer risk (Little et al., 2018; Martinez et al., 2022; Tchorz‐Trzeciakiewicz et al., 2023). Potential harmful effects of radium exposure through diet remain uncertain, however (Abergel et al., 2022; Brugge & Buchner, 2012). Although radium levels in Brazil nuts have been reported since the 1950s (Turner et al., 1958), to date, no comprehensive review of radium levels in Brazil nuts has been published.

Therefore, it was the aim of the present article to provide a global review of Ra‐226 and Ra‐288 levels in Brazil nuts published to date, including original data from Germany, and to estimate whether the observed radium levels pose a risk to consumers.

## MATERIALS AND METHODS

### Brazil nut samples (original data from Germany)

Brazil nuts (without shell) of 21 different brands (all of Bolivian origin) were purchased from the largest supermarket and drug store chains in Germany (November/December 2022). Ra‐226 and Ra‐228 (activity per mass) were assessed by gamma‐ray spectrometry (Supporting Information) at an accredited laboratory (December 2022/January 2023).

### Search strategy (literature review)

The search terms Brazil nuts OR Brazilian nuts OR nuts OR Bertholletia AND radium, radionuclides OR minerals OR elements were used in the PubMed, Web of Science and Semantic Scholar databases. The search was concluded on 31 January 2024. All titles of the indicated articles were screened to identify those which might include assessments of radium levels in Brazil nuts. Furthermore, references for identified articles were screened for additional relevant literature sources. No language restrictions were applied to the identified articles. Additional information about included and excluded publications is provided in the Supporting Information.

### Conversion factors

Published conversion factors (based on factors including type of radiation and exposure pathway) were used to convert the unit of radioactivity, Becquerel (Bq), to the unit of effective dose, Sievert (Sv): effective dose coefficients (Sv/Bq) for adults: 2.8 × 10^−7^ (Ra‐226); 6.9 × 10^−7^ (Ra‐228) (provided by the International Commission on Radiological Protection, ICRP) (Cardoso et al., 2017; Eckerman et al., 2013; Parekh et al., 2008; UNSCEAR, 2017; WHO, 2022). The effective dose is intended to be a comparable estimate of the potential health risk of ionising radiation.

### Statistical analysis

For the calculation of mean levels from the literature, the following approach was taken: if publications had subgroup‐level data available (e.g. data from different federal states or nuts purchased shelled versus in shell (Armelin et al., 2019)), each subgroup was weighted equally and included as an independent value when calculating the means (i.e. subgroup‐level means). As a sensitivity analysis, mean levels were calculated by weighting each publication equally (i.e. publication‐level means).

## RESULTS

### Publications included in the literature review

A total of 16 publications were identified that reported original data on radium levels in Brazil nuts (Ra‐226: *n* = 14; Ra‐228: *n* = 11) (Armelin, 2016; Bull et al., 2006; Gabay & Sax, 1969; Garcez et al., 2019; Hill, 1962; Hiromoto et al., 1996; Ioannidis et al., 2023; Kluczkovski et al., 2020; Kobashi & Tominaga, 1985; Martins et al., 2012; Parekh et al., 2008; Penna‐Franca, 1959; Penna‐Franca et al., 1968; Smith, 1971; Tillett et al., 2018; Turner et al., 1958). All Brazil nuts can be assumed to have originated from the Amazon rainforest, with the exception of one study in which Brazil nuts originated from a botanical garden in Singapore (Smith, 1971). No studies of Brazil nuts directly obtained in Latin American countries other than Brazil or Guyana (Smith, 1971) were identified. Two studies of Brazil nuts obtained in Asia (Singapore (Smith, 1971) and Japan (Kobashi & Tominaga, 1985)) and no studies of Brazil nuts obtained in Africa or Australia/New Zealand were identified. Mean radium (Ra‐226 and Ra‐228) levels in Brazil nuts documented in the literature are shown in Table 1.

**TABLE 1:** Radium levels in Brazil nuts from the present study and the literature.

Reference	Note regarding the nuts	Obtained in	Origin	Mean content	
				Ra‐226	Ra‐228
				mBq/g	
Present study^a^	–	Germany	Bolivia	40	43
Ioannidis et al. (2023)	–	Cyprus	Bolivia	50	–
Kluczkovski et al. (2020)	Shelled	Brazil (Óbidos, Pará)	Brazil (Óbidos, Pará)	67	69
Garcez et al. (2019)	–	Brazil (City of Rio de Janeiro)	Brazil	24	26
Tillett et al. (2018)	–	USA	Bolivia/Peru	32	–
Armelin (2016)	Shelled (2010–2013)	Brazil	Brazil	52	235
				26	186
				34	135
				65	57
Martins et al. (2012)	–	Brazil (Amazonas)	Brazil (Amazonas)	105	100
Parekh et al. (2008)	–	USA	Brazil	31	31
			Bolivia	27	22
			Peru	18	18
			Not specified	17	26
Bull et al. (2006)	–	Assumed UK	Not specified	–	12 (estimate)
Hiromoto et al. (1996)	–	Brazil	Brazil	26	31
Kobashi and Tominaga (1985)	USA import	Japan	Not specified	36	40
Smith (1971)	–	Guyana (UK study)	Guyana (Dadanawa)	24	–
			Guyana (Rewa)	[29.6–244.2]	
		Singapore (UK study)	Singapore	205	
			Singapore	30	
Gabay and Sax (1969)	Individual nuts	USA	Not specified	34	–
	~15.9 kg batch			30	
Penna‐Franca et al. (1968)	–	Brazil	Brazil	51	51
Hill (1962)	–	Assumed UK	Not specified	80	43
Penna‐Franca (1959), Penna França et al. (1967)	–	Brazil	Brazil	–	74
Turner et al. (1958)	–	UK	Not specified	67	–

^a^ Original data from Germany. Values in square brackets are provided as additional information and were not included in the calculations of means. The terms ‘in shell’ and ‘shelled’ refer to nuts being obtained (not analysed) still inside the shell or with the shell removed, respectively.

### Ra‐226 levels in Brazil nuts

The original data from Germany (Table S1) indicated a mean Ra‐226 level of 40 (range: 29–70) mBq/g, while the mean of the Ra‐226 levels reported in the literature (excluding the German data) was 49 (range: 17–205) mBq/g (median: 34 mBq/g; number of values: *n* = 23). Including the German data confirmed the results (mean: 49 mBq/g; median: 34 mBq/g, *n* = 24). Studies from the United States (US) showed a lower mean Ra‐226 level (27 [range: 17–34] mBq/g, median: 30 mBq/g, *n* = 7) than studies from Brazil (50 [range: 24–105] mBq/g, median: 51 mBq/g, *n* = 9) and Europe (UK, Cyprus and Germany: 59 [range: 40–80] mBq/g, median: 59 mBq/g, *n* = 4). In addition, one study from Japan (1980s) reported a Ra‐226 level of 36 mBq/g (Kobashi & Tominaga, 1985), while a British study (1970s) documented a lower Ra‐226 level (24 mBq/g) in Brazil nuts from Guyana (*n* = 1) but a higher level (118 mBq/g) in those from Singapore (*n* = 2) (Smith, 1971). Boxplots of Ra‐226 levels by country are shown in Figure S1. Sensitivity analyses for Ra‐226 using publication‐level means (instead of subgroup‐level means) largely confirmed the results (Supporting Information).

### Ra‐228 levels in Brazil nuts

The original data from Germany showed a mean Ra‐228 level of 43 (range: 30–65) mBq/g while the mean of the Ra‐228 levels in the literature (excluding the German data) was 68 (range: 12–235) mBq/g (median: 43 mBq/g, *n* = 17). Including the German data decreased the mean from 68 mBq/g to 67 mBq/g (median: 43 mBq/g, *n* = 18). Studies from the US indicated a lower mean Ra‐228 content (24 [range: 18–31] mBq/g, median: 24 mBq/g, *n* = 4) than studies from Europe (UK and Germany: 33 mBq/g, *n* = 3) and particularly Brazil (96 [26–235] mBq/g, median: 72 mBq/g, *n* = 10). In addition, one study from Japan (1980s) reported a Ra‐228 level of 40 mBq/g (Kobashi & Tominaga, 1985). Boxplots of Ra‐228 levels by country are shown in Figure S1. Sensitivity analyses for Ra‐228 using publication‐level means (instead of subgroup‐level means) largely confirmed the results (Supporting Information).

### Potential health risks associated with radium in Brazil nuts

The mean radium levels in Brazil nuts retrieved from the literature (including the German data) of 49 mBq/g (Ra‐226) and 67 mBq/g (Ra‐228) are equivalent to an effective dose of 0.060 μSv/g (based on both Ra‐226 and Ra‐228 levels). A daily intake of one single Brazil nut (4–5 g) would result in a radium exposure of 196–245 mBq/d (Ra‐226) and 268–335 mBq/d (Ra‐228), respectively, which can be converted to an effective dose of 0.240–0.300 μSv/d (0.055–0.069 μSv/d [Ra‐226] + 0.185–0.231 μSv/d [Ra‐228]), which in turn is equivalent to ~88–110 μSv/year. In Table 2, these effective dose values are compared to (1) limit values (i.e. exposure values that are considered acceptable for the general population), (2) average exposure values from food, (3) average exposure values from other (non‐dietary) common sources of radioactivity and (4) published risk estimates regarding exposure to radioactivity.

**TABLE 2:** Limit values, effective dose estimates, and potential health risks.

Effective dose values from consuming 1 Brazil nut (4–5 g) per day (μSv/year)	Brazil	117–146
	Germany	60–75
	Europe	57–72
	USA	35–44
	All studies	88–110
Limit values (μSv/year)		
Maximum permissible dose (limit value)	General population (BfS, 2022a; ICRP, 2019; UNSCEAR, 2000)	1000
	Occupational exposure (BfS, 2022a; ICRP, 2012, 2019; UNSCEAR, 2000)	20 000
Exposure from drinking water: value associated with a very low risk which is not expected to give rise to any detected adverse health effect (WHO, 2022)		100
Average exposure from food (μSv/year)		
Average exposure to radioactivity from natural sources among the general population (CNSC, 2022; UNSCEAR, 2000; USNRC, 2022)	Germany (USNRC, 2022)	2000–3000
	Europe (USNRC, 2022)	~3200 (range: 1500–5800)
	USA (USNRC, 2022)	~3100
	Canada (CNSC, 2022)	~1800
	Worldwide (Abergel et al., 2022; UNSCEAR, 2000; USNRC, 1999)	~2400 (range: 1000–10 000)
Estimated exposure from only natural sources in foods and drinking water (UNSCEAR, 2000)		~290–300 (range: 200–800)
Estimated exposure from only natural uranium and thorium series radionuclides (UNSCEAR, 2000)		~110–120
Estimated exposure from only Ra‐226 and Ra‐228 (UNSCEAR, 2000)	Ra‐226	~6
	Ra‐228	~10–11
Average exposure values from other common sources of ionising radiation (non‐dietary)		
Thorax X‐ray (μSv/image) (BfS, 2022a)		~10–30
Cranial CT scan (μSv/image) (BfS, 2022a)		~1000–3000
One‐way flight from Germany to Japan (μSv) (BfS, 2022a)		100
Bathing in a hot spring for 1 h (μSv) (Adelikhah et al., 2020; Radolić et al., 2005; Zdrojewicz & Strzelczyk., 2006)		~0–168 (highly variable)
Published risk estimates		
Increase in population cancer risk by 5.5% (ICRP, 2007)		Per 1 000 000 μSv
Absorbed dose level known to be harmful (Boice et al., 2019)		≥100–150 mGy (≥100 000 μSv)

*Note*: The literature values include the German data. Maximum permissible dose: limit value for any additional source of radiation exposure of members of the general public (such exposure may, e.g., result from the release of radioactive substances from nuclear facilities) (BfS, 2022a); the limit value for occupational exposure should not be used as a reference for radioactivity exposure of the general public.

Abbreviations: CT, computed tomography; mGy, milligray.

It has been proposed that each exposure increase of 1 Sv (1 000 000 μSv) would, at the population level, increase cancer risk by 5.5% (ICRP, 2007) (Table 2). Assuming that this estimate would proportionally apply to lower exposure levels as well as to radioactivity exposure from Brazil nut ingestion, a consistent daily intake of one single Brazil nut (4–5 g), equivalent to an effective dose of ~88–110 μSv/year (based on results from the literature), for a duration of 50 or 100 years (equivalent to 4400–5500 μSv or 8800–11 000 μSv, respectively) would increase population cancer risk by ~0.02%–0.03% or ~0.05%–0.06%, respectively. Thus, assuming an absolute lifetime risk of developing cancer of 40% (ACS, 2023), this risk would increase to ~40.02%–40.06%. This estimate should be considered hypothetical. There are alternative methods to calculate the change in absolute cancer risk or excess relative risk based on ICRP data (Supporting Information). However, these methods yield similar estimates (Supporting Information), which support the estimate given above (i.e. an increase to ~40.02%–40.06%).

## DISCUSSION

The original data from Germany indicated a mean Ra‐226 level of 40 mBq/g, which is similar to the mean of Ra‐226 levels described in the literature (49 mBq/g, including the German data). The mean Ra‐288 level in the nuts purchased in Germany (43 mBq/g) was lower than the mean of Ra‐228 levels reported in the literature (68 mBq/g, including the German data). Based on these radium (Ra‐226 and Ra‐228) levels, effective dose values (as a measure of potential health risk from exposure to radioactivity) of 0.041 μSv/g (Germany) and 0.060 μSv/g (literature review including the German data) were derived (UNSCEAR, 2017). Studies from Brazil showed the highest effective dose value (0.080 μSv/g).

### Radium levels in Brazil nuts previously reported in the literature

Previous studies reported effective dose values associated with radium levels in Brazil nuts that were lower (~0.020–0.030 μSv/g) (Parekh et al., 2008) or similar to those found in the present review (Hiromoto et al., 1996; Penna‐Franca et al., 1968; Rosa et al., 2022; USNRC, 2022) (Supporting Information).

### Potential reasons for the high radium levels in Brazil nuts

High radionuclide levels in the Amazon rainforest region may contribute to the high radium levels in Brazil nuts (USNRC, 1999). However, the Brazil nut tree also appears to accumulate radium (and other radionuclides) (Penna‐Franca et al., 1968; Reilly, 1999; Smith, 1971) as even Brazil nuts from locations with normal soil radium levels seem to be high in radium (Smith, 1971). Radium is chemically similar to barium and calcium (alkaline earth metals), and high groundwater barium levels may favour the transport of radium into the Brazil nut tree (USNRC, 1999). This may partly explain the co‐occurrence of relatively high levels of barium and radium in Brazil nuts (Penna‐Franca et al., 1968). Furthermore, it can be hypothesised that older Brazil nut trees will produce nuts with lower Ra‐228 levels because the half‐life of this radionuclide is relatively short (5.75 years) (Smith, 1971).

### Radium and health

No acceptable level of intake has been proposed for radium isotopes. The potential danger of low‐level exposure to radium and the question of whether a safe threshold for exposure to radioactivity exists remains controversial (Boice et al., 2019; Brugge & Buchner, 2012; WHO, 2022). Even potentially beneficial effects of low‐dose radiation exposure have been suggested (Abergel et al., 2022; Helmstädter, 2005; Iavicoli et al., 2023; Legrand et al., 2021). Natural hot springs (thermal baths), for example, can be a considerable source of exposure to radioactivity (Abergel et al., 2022; Adelikhah et al., 2020).

The main concern with exposure to ionising radiation is considered an increased cancer risk, with leukaemia being the type of cancer thought to appear earliest (Little et al., 2018). Overt harmful effects of radium have been observed in the case of occupational exposure, one of the most well‐known historical examples being that of the radium dial workers in the US and Canada (Brugge & Buchner, 2012; Martinez et al., 2022). In the first half of the 20th century, radium watch‐dial painters, mostly young women, unknowingly ingested extremely high quantities of radium from radioluminescent paint. Many women were instructed to give the paintbrushes a fine point using their lips (Loutit, 1970; Martinez et al., 2022). After several years, many of the workers developed health problems, including toothaches, mouth sores, painful gums and jaws, tooth loss, bone fractures and bone cancer (including mastoid and paranasal sinus carcinomas) (Loutit, 1970; Martinez et al., 2022; Roberts, 2017). In these dial painters, oral radium exposure was extremely high. It has been suggested that they handled 50–500 μg of radium (Ra‐226 and Ra‐228) on a daily basis (radioactivity: ~2–19 billion mBq) (Martinez et al., 2022) (i.e. ~3–95 million times the amount in a typical Brazil nut [on average ~200–700 mBq/nut; Table 1]), although the watch‐dial painters only ingested a fraction of the radium they handled. In the radium dial painters, no bone cancer was reportedly observed in those with an estimated radium ingestion below 100 μCi (3.7 billion mBq) (Martinez et al., 2022). Moreover, in those radium dial painters whose radium intake was primarily Ra‐228, no mastoid and paranasal sinus carcinomas were observed (Martinez et al., 2022). Nevertheless, many of the radium watch‐dial painters appear to have died at a young age from radioactivity‐induced causes other than cancer (e.g. anaemia or bone necrosis and related infections (Loutit, 1970; Martinez et al., 2022)). Other cases of very high radium exposure in humans have been described (Brugge & Buchner, 2012; Loutit, 1970; Monged, 2020).

In some countries, there are upper levels for caesium‐137 (an artificial radionuclide) in foods (BfS, 2022b; Osanai et al., 2021) but no such upper levels appear to have been set for natural radionuclides in foods. Nevertheless, the German Nutrition Society (DGE) recommends eating no more than one or two Brazil nuts per day, due to the levels of radioactivity in these nuts (DGE, 2024). In addition, the Federal Office for Radiation Protection (BfS) in Germany states that Brazil nuts should not be eaten during childhood, adolescence, pregnancy and breastfeeding because radium may be deposited in the bones and unnecessary radiation should be avoided (BfS, 2024).

A hypothesised upper percentile consumer with a consistent Brazil nut intake of, for example, 50 g/d (assuming 0.060 μSv/g for Brazil nuts) would be exposed to an effective dose of ~1095 μSv/year, which would exceed the limit value of 1000 μSv/year (BfR, 2021; ICRP, 2019). However, this would still be below the average effective dose of ~2400 μSv/year resulting from natural sources among the general population (UNSCEAR, 2000; USNRC, 1999).

It has also been suggested that foods high in radium often also contain high levels of other alkaline earth metals (i.e. barium and strontium), which are chemically similar, one being able to substitute the other in plant and human tissues (Mayneord, 1960). While most foods appear to contain much lower levels of radioactivity than Brazil nuts (Ghajarbeygi et al., 2023; Rayman, 2008; Sadighara et al., 2023), some foods may have radionuclide levels comparable to those found in Brazil nuts. Some seafood, for example, is known to contain high levels of polonium (Po‐210). A study from Italy found a Po‐210 content of 33 mBq/g cooked European anchovy (*Engraulis encrasicolus*), which is equivalent (conversion factor [Sv/Bq]: 1.2 × 10^−6^) (UNSCEAR, 2017) to an effective dose of ~1.98 μSv per 50 g portion (a realistic serving size) (Roselli et al., 2020). This in turn is comparable to (and higher than) the effective dose of 0.48–0.60 μSv per two Brazil nuts (8–10 g), which is indicated by the results of the present review. Other studies confirm similar or higher levels of Po‐210 in anchovies. A study from Turkey found an approximately four times higher Po‐210 content in European anchovies (~7.08 μSv per 50 g raw anchovy) (Sezer et al., 2023) compared to the Italian study (Roselli et al., 2020). A study from Korea found two samples of Japanese anchovy (*Engraulis japonicus*) to contain about twice and 12 times as much Po‐210 (~3.54 and ~23.52 μSv per 50 g raw anchovy) (Kim et al., 2017) as the Italian study. Even foods with considerably lower levels of radioactivity than Brazil nuts can lead to similar exposure levels if the intake of these foods is high (Silva et al., 2007). Similarly, radionuclide levels in drinking water may also result in a comparable effective dose (e.g. ~0.05 μSv/L (Beyer et al., 2020)) to the one observed in Brazil nuts (~0.10–0.40 μSv/Brazil nut).

The permissible upper level for the sum of Ra‐226 and Ra‐228 in drinking water in the US is 185 mBq/L (5 pCi/L) which is equivalent to an effective dose of ~0.05–0.13 μSv/L (ATSDR, 2014). Thus, with a water intake of ~2–4 L/d (Seal et al., 2023) the effective dose from drinking water may exceed that associated with a daily Brazil nut if radioactivity levels in water are high but below the upper level (5 pCi/L (ATSDR, 2014)). In addition, actual radioactivity levels in drinking water are not always below this permissible upper level (USNRC, 1999). Furthermore, the WHO suggests that drinking water levels of 500 mBq/L for gross alpha activity and 1000 mBq/L for gross beta activity are safe (WHO, 2022).

It should be noted that while the conversion of Bq (unit of radioactivity) to Sv (unit of effective dose) aims to take into account the factors of radiation type, exposure pathway, and body region affected (WHO, 2022), the suggested conversion factors may insufficiently consider the factor of bioavailability in the intestine. This may be highly relevant when it comes to the ingestion of radionuclides from food because, if bioavailability is low (Brugge & Buchner, 2012), the conversion factors used above would likely overestimate the effective dose (in Sv) associated with a given quantity of radioactivity (in Bq). Radium sulphate is relatively insoluble in water (USNRC, 1999), and it has been suggested that only a small amount of the radium present in Brazil nuts is absorbed in the human intestine (Brugge & Buchner, 2012; Gabay & Sax, 1969; Mayneord, 1960; Rayman, 2008). Similarly, it has been suggested that intestinal radium absorption in general is low (Brugge & Buchner, 2012) and that ~80% of the radium ingested with food is rapidly excreted in faeces, whereas the remaining 20% will enter the circulation from where this fraction will be excreted (via faeces and urine) or permanently deposited in the bones (ATSDR and US DHH, 1990; Mayneord, 1960; US EPA, 2023). It has been reported that ~0%–25% of the radium ingested is retained in the body although interpersonal variability may be large (Brugge & Buchner, 2012; Mayneord, 1960). In the 1960s, it was also suggested that some of the ingested radium is deposited in soft tissues (Mayneord, 1960) and that other factors such as the high barium levels in Brazil nuts, calcium balance and vitamin D intake may affect intestinal absorption of radium from Brazil nuts (Hallden & Harley, 1964; Penna‐Franca et al., 1968). However, a study from the 1970s in which adult men (*n* = 15) in a metabolic research unit ingested Ra‐226 (~25–33 mBq/d) naturally present in food, calcium intake (in a range of 170–3030 mg/d) did not seem to affect Ra‐226 deposition in the bones (Spencer et al., 1973). However, no similar data are available for children or pregnant women. As such, a precautionary recommendation for children and pregnant women to avoid Brazil nuts (as suggested by the BfS in Germany (BfS, 2024)) might be justified.

While some suggest that a linear no‐threshold model (assuming that no dose is entirely safe) may overestimate potential harm from low‐dose radiation exposure (Iavicoli et al., 2023), others suggest that the established conversion factors (Eckerman et al., 2013) may, in some cases, considerably underestimate adverse effects of low‐dose radiation, citing evidence that low‐dose radioactivity exposure, which may be considered safe (e.g. <1000 μSv/year (ICRP, 2019)) based on established conversion factors (Eckerman et al., 2013), has not always been found to be safe (Busby, 2022). It has furthermore been suggested that alkaline earth metals other than calcium (i.e. beryllium, magnesium, strontium, barium and radium) might, due to their chemical similarity to calcium, replace calcium in binding to the DNA phosphate backbone and that radioactive isotopes of these metals might therefore be of particular concern (Busby, 2022). Any such adverse effects may not just translate into a higher risk for cancer but also for other chronic diseases (Busby, 2022). In contrast, some studies even indicate a potential protective effect of low‐dose radiation, in line with the concept of hormesis, which states that some substances may be beneficial at low doses but toxic at higher doses (Iavicoli et al., 2023). Moreover, the concept of a hormetic preconditioning dose has been proposed according to which exposure to low‐dose radioactivity may stimulate enhanced DNA repair (Calabrese, 2024) and may protect against future higher‐dose exposure (Busby, 2022; Iavicoli et al., 2023). Therefore, while the effective dose values and comparisons with upper levels presented in the present paper (Table 2) are useful, they are based on the crude assumption of the accuracy of established conversion factors.

### Future research

Randomised controlled trials (RCTs) assessing the effect of a regular Brazil nut intake on signs of anaemia, markers of cancer risk and other health markers would be useful. Potential biomarkers to assess may include circulating soluble tumour necrosis factor receptor‐2 (Epstein et al., 2018), C‐reactive protein, insulin‐like growth factor‐1 and lactate dehydrogenase (for cancer in general) (Desai & Guddati, 2023; Feng et al., 2018; Zhang et al., 2024), testosterone and prolactin (in women, for breast cancer) (Aranha et al., 2022; Zhang et al., 2024), and prostate‐specific antigen and novel associated markers (Garrido et al., 2022) as well as coagulation markers (Lei et al., 2023) (in men, for prostate cancer). Nevertheless, to date, many cancer markers still have a weak positive predictive value (Desai & Guddati, 2023). Due to the established association between radioactivity exposure and leukaemia (Little et al., 2018; Loutit, 1970), biomarkers of particular relevance to leukaemia should be assessed (Epstein et al., 2018). However, all types of cancer are relevant in connection with exposure to radioactivity (ICRP, 2007; Loutit, 1970).

Due to the unique nutritional profile and the relative popularity of Brazil nuts, researchers conducting large observational studies should consider assessing the intake of Brazil nuts. Furthermore, case reports of upper percentile consumers of Brazil nuts in which any signs of aplastic/leucopenic anaemia and elevation of cancer risk biomarkers are assessed may be a possibility.

### Strengths and limitations

The present article appears to be the first global overview of radium in Brazil nuts and the first to include data from Germany. However, the present analysis cannot claim to be a complete overview of all radium levels in Brazil nuts ever published, and it was beyond the scope of the present review to screen the entire (extensive) literature (including books and grey literature) on Brazil nuts in Portuguese (EMBRAPA, Coutinho Vaz Pereira, and Lima Costa, 1981). Nevertheless, the results appear to be representative, and the mean values documented since the 1950s appear surprisingly similar. A limitation of the original data from Germany is that samples were analysed without repeat measurements, but variability was relatively small (Table S1). It should be noted that the effective dose values estimated in the present article are based on Ra‐226 and Ra‐228 levels only and thus underestimate the effective dose based on total radionuclide content of Brazil nuts (Brugge & Buchner, 2012; Bull et al., 2006). A detailed look at radium and other radionuclides in foods or dietary patterns in general (Kaur et al., 2020; Shiraishi et al., 1994; USNRC, 1999) was beyond the scope of the present analysis. Moreover, the risk assessment in the present paper is based on conversion factors proposed by ICRP, which are not universally accepted (Busby, 2022).

## CONCLUSIONS

The Federal Office for Radiation Protection in Germany recommends that children, adolescents, pregnant women, and breastfeeding women avoid eating Brazil nuts as a precautionary measure. It appears uncertain whether this recommendation is justified. The present review found that a consistent long‐term intake of one Brazil nut per day results in a radioactivity exposure of approximately 100 μSv/year (based on Ra‐226 and Ra‐228 levels), which appears to be comparable to radioactivity exposure from drinking water (~100 μSv/year). It has been proposed that such low‐level radioactivity exposure should not be expected to result in any adverse health effects. Nevertheless, the discussion about whether there can be a safe threshold for radioactivity exposure or whether absolute minimisation of this exposure should be the strategy is ongoing. Consistently consuming slightly higher quantities (e.g. 10 Brazil nuts/day) can easily exceed the maximum permissible dose (limit value) of 1000 μSv/year suggested by health authorities in the field (e.g. the United Nations Scientific Committee on the Effects of Atomic Radiation and ICRP). While any potential health risk that may arise from the radioactivity in Brazil nuts should not be ignored and statements that these levels are clearly harmless may be difficult to justify, the potential risk should not be exaggerated. Scientists should discourage a reductionist approach to assessing and reporting the potential health effects of foods as well as any sensationalising of study results, particularly as radioactivity exposure may be easily misinterpreted by the general public. Furthermore, the complex sustainability aspects of this food should be taken into account. Human intervention studies could bring more clarity regarding the health effects of Brazil nuts.

## AUTHOR CONTRIBUTIONS

C.K.: methodology, conceptualisation, writing – original draft, and writing – review and editing. M.K.: methodology, conceptualisation, writing – review and editing.

## FUNDING INFORMATION

No funding was received for writing this article.

## CONFLICT OF INTEREST STATEMENT

The authors declare that they have no competing interests.

## Supporting information


**File S1.**.

## Data Availability

All data are included in the present review.
