# Supplementary material for: Radium levels in Brazil nuts: A review of the literature
Source: Nutr Bull. 2024 Nov 3;50(1):1–11. doi: 10.1111/nbu.12717 (PMC11815606; doi:10.1111/nbu.12717)
Supplement: Supplementary file 1 — File S1.. [file NBU-50-1-s001.docx]

*Supplementary material*

*Radium levels in Brazil nuts: a review of the literature*

*Christian Koeder and Markus Keller*

# Materials and methods

## **Sample analysis of Brazil nuts purchased in Germany (original data)**

Ra-226 and Ra-228 (activity per mass) were assessed by gamma-ray spectrometry at an accredited laboratory (IAF-Radioökologie GmbH, Radeberg, Germany). The laboratory received one packet of each brand of Brazil nuts. The decision thresholds and detection limits were 1 mBq/g and 2 mBq/g for Ra-226 and 1.5 mBq/g and 3 mBq/g for Ra-228, respectively. Measured values above the detection limit can be reliably reproduced. The samples were analysed on behalf of the consumer magazine ÖKO-TEST (Frankfurt am Main, Germany). Due to limited funding, radium levels were analysed without repeat measurements.

## **Additional comments regarding Table 1: publications included and excluded**

| **Included in the present article:**  -- Tillett et al. 2018 (DOI: 10.1016/j.apradiso.2018.08.013) reported a Ra-226 value of 33 mBq/g dry weight which can be converted to ~32 mBq/g fresh weight (based on an assumption of 3.4% water content in Brazil nuts [USDA 2019: https://fdc.nal.usda.gov/fdc-app.html#/food-details/170569/nutrients]).  -- Parekh et al. 2008 (DOI: 10.1016/j.jfca.2007.12.001) reported radium values for 4 locations: Brazil, Bolivia, Peru as well as an unknown location. This fourth sample of Brazil nuts (unknown location) was labelled ‘‘Product of the USA’’ but as there is no commercial production of Brazil nuts in the United States, Parekh et al. (reasonably) assumed that this must have been a labelling error. In their article, they referred to the origin of this fourth sample as northern South America (NSA). It can be reasonably assumed that practically all commercially available Brazil nuts originate from the Amazon rainforest, which is located in the northern (i.e., the “northern half” of) South America. Several authors citing Parekh et al. have misinterpreted ‘‘northern South America’’ to mean probably Colombia or Venezuela (i.e., the very north of South America).  -- Bull et al. 2006 (DOI: 10.1093/rpd/ncl059) did not assess Ra-226 or Ra-228 values but suggested that “If secular equilibrium in the Brazil nut is assumed then the concentration of 228Ra would be equal to that for 228Th given in Table 2”, i.e., 11.9 mBq/g. This value was included in Table 2 of the present article.  -- Kobashi & Tominaga 1985 (DOI: 10.1016/0020-708X(85)90108-5) reported Ra-226 and Ra-228 in dpm (disintegrations per minute). We converted the reported values. Ra-226: 2.23 dpm/g (dry weight) = 2.15 dpm/g (fresh weight) [based on an assumption of 3.4% water content in Brazil nuts [USDA 2019: https://fdc.nal.usda.gov/fdc-app.html#/food-details/170569/nutrients)] = 35.90 mBq, which equivalent to 0.010 µSv; Ra-228: 2.49 dpm/g (dry weight) = 2.41 dpm/g (fresh weight) = 40.09 mBq, which is equivalent to 0.028 µSv.  -- Smith 1971 (DOI: 10.1007/BF01372791) reported Ra-226 values in pCi/g which we converted to mBq/g: Guyana (Dadanawa): 0.660 pCi/g (24 mBq/g), Singapore: 5.53 pCi/g (205 mBq/g), Singapore: 0.80 pCi/g (30 mBq/g). For Guyana (Rewa), only a range was reported by Smith (0.80–6.60 pCi/g [29.6–244.2 mBq/g]), and we did not include this value in our calculations. Smith 1971 estimated that the Ra-228 values may be 3.32 times as high as the Ra-226 values (we did not include Ra-228 values based on this estimate in the present article).  -- Gabay & Sax 1969 (see references) reported data from a conference presentation by Gaby & Sax 1963; they reported the Ra-226 values in pCi/g which we converted to mBq/g (for individual kernels: 0.930 pCi/g [34 mBq/g]; for a 35 lb. [~15.9 kg] batch: 0.820 pCi/g [30 mBq/g]).  -- Penna França et al. 1968 reported the radium levels in pCi/g which we converted to mBq/g: Ra-226: 1.38 pCi/g (51 mBq/g), Ra-228: 1.37 pCi/g (51 mBq/g).  -- Hill 1962 (DOI: 10.1097/00004032-196202000-00003) reported radium levels in pCi/g (“µµc/g”) which we converted to mBq/g: 2.15 pCi/g (80 mBq/g) and 1.15 pCi/g (43 mBq/g).  -- Penna França 1959: the Ra-228 values reported by Penna França 1959 are cited from Penna França et al. 1968 (see references) and are also documented in Penna França et al. 1967 (‘Radioatividade Das Castanhas Do Pará’, see above). Penna França 1959 reported the Ra-228 values in pCi/g, which we converted to mBq/g: 2.00 pCi/g (74 mBq/g).  -- Turner et al. 1958 (DOI: 10.1097/00004032-195807000-00002): the mean Ra-226 value (1.80 pCi/g [67 mBq/g]) reported by Turner et al. in Table 2 of the present article is cited from Gabay & Sax 1969 (see references). Only the maximum Ra-226 value is found in Turner et al. 1958 (2.73 pCi/g [101 mBq/g]). |
| --- |
| **Excluded from the present article:**  -- Macan et al. 2020 (DOI: 10.1080/01480545.2020.1808667) did not report original data regarding radium levels in Brazil nuts.  -- Klement 2018 (CRC Handbook of Environmental Radiation) did not report original data regarding radium levels in Brazil nuts.  -- Kritzberger & Navarrete 2017 (DOI: 10.2967/jnmt.117.196451) did not report radium levels in Brazil nuts.  -- da Silva 2014 (master thesis, Brazil, DOI: 10.11606/D.11.2014.tde-05012015-113355) did not report radium levels in Brazil nuts.  -- Kannamkumarath et al. 2004 (DOI: 10.1021/jf0496649) did not report radium levels in Brazil nuts.  -- Maul & O'Hara 1989 (DOI: 10.1016/0265-931X(89)90048-9) did not report original data regarding radium levels in Brazil nuts.  -- Penna França et al. 1967 [E. Penna-Franca; M. Fiszman; N. Lobao; C. Costa Ribeiro; H. A. Trinidade; P. L. Dos Santos: Radioatividade das castanhas do Para, Atas do Simposio sobre a biota amazonica, Vol. 4 (Botanica), 187–208 (1967)]: the values reported by Penna França et al. 1967 (their *Tabela I* and *Tabela II*) are also reported in Penna França et al. 1968 [https://journals.lww.com/health-physics/Abstract/1968/02000/Radioactivity_of_Brazil_Nuts.2.aspx].  -- Turner 1962 (DOI: 10.1038/bjc.1962.23) did not report original data regarding radium in Brazil nuts but cites Turner et al. 1958 (DOI: 10.1097/00004032-195807000-00002) and Mayneord 1960 (DOI: 10.1016/S0009-9260(60)80059-1).  -- Straub et al. 1961 (DOI: 10.1016/S0002-8223(21)23061-7) did not report radium levels in Brazil nuts.  -- Mayneord et al. 1960 (DOI: 10.1038/187208a0) reported no data regarding radium in Brazil nuts.  -- Leonardos 1958 (Leonardos, Othon: Sobre a radioatividade das castanhas do Para, Annaes da Academia Brasileira de Ciencia, Volume 30(4), page LI-LII. 1958; http://memoria.bn.br/DocReader/DocReader.aspx?bib=158119&Pesq=leonardos&pagfis=11661; last accessed 28 February 2024) reported no original radium levels in Brazil nuts. |

# Results

## **Supplementary table 1**

| **Supplementary table 1. Radium levels in Brazil nuts purchased in Germany (original data)** | | | |
| --- | --- | --- | --- |
| **Brazil nut brands** | **Organic**  **(yes/no)** | **Ra-226** | **Ra-228** |
|  |  | **mBq/g** | **mBq/g** |
| Sample 1 | No | 50 | 59 |
| Sample 2 | Yes | 45 | 50 |
| Sample 3 | No | 36 | 37 |
| Sample 4 | No | 41 | 47 |
| Sample 5 | Yes | 39 | 42 |
| Sample 6 | Yes | 37 | 33 |
| Sample 7 | Yes | 45 | 50 |
| Sample 8 | Yes | 34 | 39 |
| Sample 9 | No | 38 | 41 |
| Sample 10 | Yes | 43 | 47 |
| Sample 11 | No | 37 | 39 |
| Sample 12 | No | 37 | 43 |
| Sample 13 | No | 37 | 43 |
| Sample 14 | No | 33 | 34 |
| Sample 15 | No | 29 | 30 |
| Sample 16 | No | 39 | 35 |
| Sample 17 | Yes | 70 | 65 |
| Sample 18 | Yes | 39 | 33 |
| Sample 19 | No | 46 | 43 |
| Sample 20 | No | 38 | 40 |
| Sample 21 | No | 37 | 44 |
| Mean | - | 40 | 43 |
| Median | - | 38 | 42 |
| Range | - | 29–70 | 30–65 |

## **Supplementary figure 1**


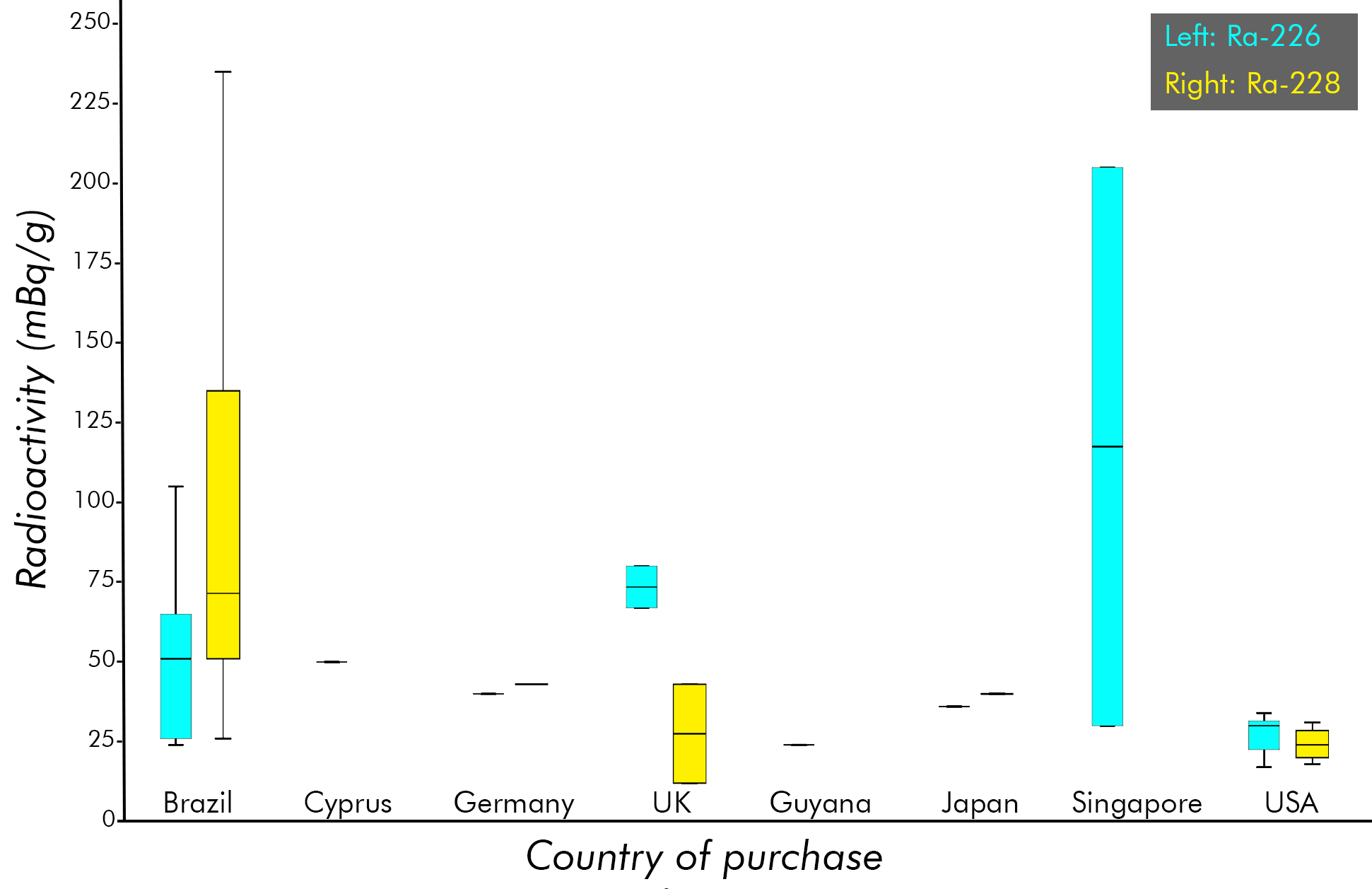


**Supplementary figure 1. Radioactivity levels in Brazil nuts by country of purchase**. Left (blue): Ra-226; right (yellow): Ra-228. For Cyprus, Guyana, and Singapore only Ra-226 values were available.

## **Sensitivity analyses for Ra-226 using publication-level means (instead of subgroup-level means)**

Sensitivity analyses for Ra-226 using publication-level means (instead of subgroup-level means) largely confirmed the results. Mean Ra-226 levels slightly increased from 49 to 52 mBq/g (all studies, excluding the German data, n = 15), from 49 to 51 mBq/g (all studies, including the German data, n = 16), from 27 to 29 mBq/g (USA, n = 3), and from 50 to 53 mBq/g (Brazil, median: 48 mBq/g, n = 6), with no changes for Europe.

## **Sensitivity analyses for Ra-228 using publication-level means (instead of subgroup-level means)**

Sensitivity analyses for Ra-228 using publication-level means (instead of subgroup-level means) largely confirmed the results: mean Ra-228 levels decreased from 68 to 57 mBq/g (all studies, excluding the German data, median: 43 mBq/g, n = 11), from 67 to 56 mBq/g (all studies, including the German data, median: 43 mBq/g, n = 12), and from 96 to 72 mBq/g (Brazil, median: 69 mBq/g, n = 7), with no changes for the US (n = 1) and Europe (n = 3).

## **Alternative methods to calculate the change in absolute cancer risk or excess relative risk**

Based on ICRP publication 103 (ICRP, 2007. The 2007 Recommendations of the International Commission on Radiological Protection. ICRP Publication 103. Ann. ICRP 37 (2-4).),Table A.4.6 Coefficients in the current cancer incidence-based ERR models", an excess relative risk (ERR) of ~50% per Gray (Gy) may be assumed:

Table A.4.6. (ICPR 103) lists a range of ERR coefficients for solid [!] tumours (i.e., excluding non-solid cancer). The ERR coefficients listed for all solid tumours are 0.35 for men and 0.58 for women, resulting in an average ERR coefficient (assuming 50% men and 50% women) of 0.465. Assuming that solid cancers make up ~93% of all cancer (based on Tables A.4.10 and A.4.11 at age 70 years, ICPR 103), this coefficient should be adjusted from 0.465 to 0.500 (= 50%).

-- Assuming a conversion rate of 1 Gy = 1 Sv and the same assumptions given in the main text (an effective dose of 4,400–5,500 µSv or 8,800–11,000 µSv for a duration of 50 or 100 years, respectively), one obtains an ERR of 0.22–0.28% (50 years) or 0.44–0.55% (100 years). This would lead to an increase in absolute lifetime cancer risk from 40% to 40.09–40.22% (compared to the estimate of 40.02–40.06% given in the main text). However, this higher estimate appears to be an overestimation because Table A.4.6 provides "ERR per Gy [Gray] at age 70 for exposure at age 30". The assumption of 1 Gy = 1 Sv is adequate for beta radiation but not alpha radiation. For the latter a conversion of 1 Gy = 20 Sv is more adequate.

-- The present analysis (main text) showed that Ra-226 constituted 25.2% of the total Ra (Ra-226 + Ra-228; data not shown). Ra-226 emits practically only alpha radiation and Ra-228 emits only beta radiation. Therefore, a combined conversion rate for both Ra-226 (25.2%) and Ra-228 (74.8%) can be derived: 1 Gy = 20 Sv * 0.252 + 1 Sv * 0,748 = 5.788 Sv. This results in an ERR coefficient of ~8.6% (ERR per 1 Sv). This in turn leads to an increase in absolute lifetime cancer risk from 40% to 40.02–40.04% (compared to 40.02–40.06% given in the main text).

**Discussion**

**Radium levels in Brazil nuts previously reported in the literature**

Hiromoto et al. (Brazil, 1990s) estimated (based on Ra-226, Ra-228, U-238, Th-232, and Th-228) that a long-term Brazil nut intake of 100 g/week would amount to an effective dose of ~200 µSv/year, i.e., 0.038 µSv per gram of Brazil nuts (Hiromoto et al. 1996). Even though they took other radioactive isotopes into account, these effective dose values are slightly lower than the ones documented in the present article, i.e., 0.041 µSv/g (Germany), 0.060 µSv/g (studies worldwide), and 0.080 µSv/g (Brazil). Parekh et al. (2008) published effective dose estimates for Brazil nuts (based on Ra-226 and Ra-228) of ~0.017 µSv/g (Peru), ~0.023 µSv/g (Bolivia), and ~0.030 µSv/g (Brazil) (Parekh et al. 2008). Similarly, Penna Franca et al. (1968) reported radioactivity levels in Brazil nuts of ~53 mBq/g (~1.42 pCi/g) for each Ra-226 and Ra-228 (Penna-Franca et al. 1968), which is equivalent to an effective dose (from Ra-266 + Ra-228) of ~0.051 µSv/g. The US Nuclear Regulatory Commission (USNRC) has suggested radioactivity levels in Brazil nuts of 37–259 mBq/g (1–7 pCi/g; based on Ra-226) (USNRC 2022), i.e., 0.010–0.073 µSv/g. As such, previously reported values are comparable to the values found in the present analysis (**Table 2**). Furthermore, Rosa et al. (2022), while not documenting exact values for Ra-226 and Ra-228 in Brazil nuts from a high background radiation area in Brazil, reported radioactivity levels of “tens” of mBq/g for Ra-226 and “hundreds” of mBq/g for Ra-228 (Rosa et al. 2022), which is similar to the mean Ra-226 level we found (49 [range: 17–205] mBq/g, based on all studies) but higher than the mean Ra-228 level we observed (68 [range: 12–235] mBq/g, based on all studies).
